# Hippocampal glial inflammatory markers are differentially altered in a novel mouse model of perimenopausal cerebral amyloid angiopathy

**DOI:** 10.3389/fnagi.2023.1280218

**Published:** 2023-11-15

**Authors:** Jimcy Platholi, Roberta Marongiu, Laibaik Park, Fangmin Yu, Garrett Sommer, Rena Weinberger, William Tower, Teresa A. Milner, Michael J. Glass

**Affiliations:** ^1^Weill Cornell Medicine, Feil Family Brain and Mind Research Institute, New York, NY, United States; ^2^Anesthesiology Department, Weill Cornell Medicine, New York, NY, United States; ^3^Neurological Surgery Department, Weill Cornell Medicine, New York, NY, United States; ^4^Genetic Medicine Department, Weill Cornell Medicine, New York, NY, United States; ^5^Aligning Science Across Parkinson’s (ASAP) Collaborative Research Network, Chevy Chase, MD, United States; ^6^Harold and Milliken Hatch Laboratory of Neuroendocrinology, The Rockefeller University, New York, NY, United States

**Keywords:** amyloid beta, microglia, astrocytes, blood vessel, subiculum, aging model

## Abstract

Dementia is often characterized by age-dependent cerebrovascular pathology, neuroinflammation, and cognitive deficits with notable sex differences in risk, disease onset, progression and severity. Women bear a disproportionate burden of dementia, and the onset of menopause (i.e., perimenopause) may be a critical period conferring increased susceptibility. However, the contribution of early ovarian decline to the neuroinflammatory processes associated with cerebrovascular dementia risks, particularly at the initial stages of pathology that may be more amenable to proactive intervention, is unknown. To better understand the influence of early ovarian failure on dementia-associated neuroinflammation we developed a model of perimenopausal cerebral amyloid angiopathy (CAA), an important contributor to dementia. For this, accelerated ovarian failure (AOF) was induced by 4-vinylcyclohexene diepoxide (VCD) treatment to isolate early-stage ovarian failure comparable to human perimenopause (termed “peri-AOF”) in transgenic SWDI mice expressing human vasculotropic mutant amyloid beta (Aβ) precursor protein, that were also tested at an early stage of amyloidosis. We found that peri-AOF SWDI mice showed increased astrocyte activation accompanied by elevated Aβ in select regions of the hippocampus, a brain system involved in learning and memory that is severely impacted during dementia. However, although SWDI mice showed signs of increased hippocampal microglial activation and impaired cognitive function, this was not further affected by peri-AOF. In sum, these results suggest that elevated dysfunction of key elements of the neurovascular unit in select hippocampal regions characterizes the brain pathology of mice at early stages of both CAA and AOF. However, neurovascular unit pathology may not yet have passed a threshold that leads to further behavioral compromise at these early periods of cerebral amyloidosis and ovarian failure. These results are consistent with the hypothesis that the hormonal dysregulation associated with perimenopause onset represents a stage of emerging vulnerability to dementia-associated neuropathology, thus providing a selective window of opportunity for therapeutic intervention prior to the development of advanced pathology that has proven difficult to repair or reverse.

## 1 Introduction

Dementia is characterized by a progressive impairment in cognitive function leading to an inability to independently carry out daily activities (Arvanitakis et al., 2019). The mechanisms subserving dementia include neurodegenerative processes, oxidative stress, neuroinflammation characterized by the induction of reactive astrocytes and microglia, as well as cerebrovascular dysfunction (Raz et al., 2016). Indeed, macro and micro infarcts, intraparenchymal hemorrhage, atherosclerosis, and arteriosclerosis are associated with dementia risk (Snyder et al., 2015). Additionally, cerebral amyloid angiopathy (CAA), characterized by the aggregation of amyloid-beta (Aβ) in arterioles and small vessels, alone, or in conjunction with parenchymal Aβ plaque formation as seen in Alzheimer’s disease (Malhotra et al., 2022), is an important form of cerebrovascular pathology associated with dementia.

Microvascular disease (Patel et al., 2020), including CAA (Masuda et al., 1988), may be more prevalent in women, with increased risk for vascular dysfunction specifically arising at menopause. Perimenopause, the transitional state of irregular gonadal hormonal cycling prior to full menopause (i.e., postmenopause), may be a particularly vulnerable time for the emergence of vascular disease (Moreau, 1985; McCarthy and Raval, 2020; Nair et al., 2021) and cerebral Aβ aggregation in females (Mosconi et al., 2017, 2018). However, the mechanisms underlying the increased risk for cerebrovascular impairment, neuroinflammation, and cognitive decline at perimenopause are unclear.

Alterations in gonadal hormone production and cycling at the onset of menopause may be expected to play a role in the increased susceptibility to dementia in women. For example, reproductive factors that are associated with low lifelong cumulative exposure to endogenous estrogen are linked with increased dementia risk (Gong et al., 2022). Further, earlier age at menopause is associated with an elevated risk of incident dementia (Liao et al., 2023). Brain pathology characteristic of dementia may emerge early in menopause as shown by a perimenopausal decline in the volume of the hippocampus, a brain area critical for learning and memory (Mosconi et al., 2018); increased Aβ expression (Mosconi et al., 2017, 2018) and impaired neurovascular integrity (Snyder et al., 2015; Jayachandran et al., 2021) are also observed at perimenopause. More controversially, estrogen replacement initiated soon after menopause onset may attenuate signs of dementia (Mills et al., 2023). Despite the apparent importance of perimenopause in the emergence of dementia, the contribution of perimenopausal hormonal changes to key neuroinflammatory and dementia-related vascular pathologies, particularly cerebral amyloidosis, is unclear.

Advances in our understanding of perimenopausal susceptibility to CAA are likely to emerge from appropriate mouse models. Transgenic mice expressing human vasculotropic mutant Aβ precursor protein (transgenic SWDI mice) recapitulate select features of human CAA. These mice express heavy vascular Aβ deposition, as well as diffuse parenchymal amyloid aggregation, particularly in the hippocampus (Xu et al., 2007, 2014). Additionally, male SWDI mice show microvascular amyloid-associated impairments in spatial learning and memory as early as 3 months of age, as well as localized neuroinflammation that further drives disease pathophysiology (Thal et al., 2003; Harkness et al., 2004; Miao et al., 2005b; Jäkel et al., 2017). Significantly, the elevated Aβ in SWDI mice is further associated with activation of glial cells that coordinate neurovascular signaling. These include neuroinflammatory reactive astrocytes and activated microglial (Miao et al., 2005b) with severity directly correlated with the number of mutant alleles (Xu et al., 2007).

Combining a rodent model of menopause with transgenic mouse dementia models to specifically isolate the influence of perimenopausal hormonal changes in dementia-associated neuropathology is problematic. The traditional experimental approach to study menopause in rodents involves performing ovariectomy, but this approach produces immediate cessation of ovarian hormones (i.e., surgical but not natural menopause) and therefore does not recapitulate the changes in gonadal hormone production and cycling characteristic of perimenopause (Van Kempen et al., 2011; Marques-Lopes et al., 2018). Another common strategy, natural aging, lacks a specific perimenopause stage, confounds chronological and ovarian aging, and thus is also inappropriate for the study of hormone changes occurring during perimenopause (Van Kempen et al., 2011; Marques-Lopes et al., 2018). A more recent but less widely used method is to administer ovotoxin 4-vinylcyclohexene diepoxide (VCD), which results in a form of accelerated ovarian failure (AOF) that mimics the extended hormone cycles characteristic of early human menopause (termed “peri-AOF”), before transitioning to an advanced stage that parallels full menopause (Van Kempen et al., 2014; Brooks et al., 2016; Marques-Lopes et al., 2017). Additionally, AOF can be initiated at different points in adult development including early adulthood, thus eliminating the confound of aging to selectively target altered hormonal signaling (Van Kempen et al., 2014; Brooks et al., 2016; Marques-Lopes et al., 2017).

Using the AOF method, we generated the first perimenopausal model of CAA utilizing SWDI mice. It is important to note that the body of basic and preclinical dementia research is weighted toward the study of advanced disease models. However, reversal of neurodegenerative processes in the clinic has generally proven difficult at late disease stages. Significantly, recent data suggests that early hormone replacement at perimenopause may have beneficial effects on neurocognitive decline compared to later treatment (Sung et al., 2022; Saleh et al., 2023). In this light, investigating disease development, as opposed to established disease, may lead to preventive or disease-arresting therapeutic strategies with improved outcomes for menopausal women. Thus, in the present study experimental conditions were limited to the early stages of ovarian failure and amyloidosis to identify the earliest manifestations of pathology, a strategy that may help to identify novel indicators (e.g., biomarkers) and targets (e.g., interventions) of disease development and progression. In this context, we investigated the hypothesis that the hormonal dysregulation characteristic of an early stage of accelerated ovarian failure and CAA independent of aging will promote neuroinflammation in the hippocampus of mice expressing human mutant Aβ precursor protein.

## 2 Experimental procedures

### 2.1 Animals

Young adult (~2 month-old at the initiation of the experiments; Flurkey and Currer, 2004) female C57BL/6 [wild type (WT); *N* = 22] and transgenic SWDI (C57BL/6 background; *N* = 22) mice were bred and maintained in a colony at Weill Cornell Medicine (WCM). Breeding pairs of SWDI mice were obtained from the Jackson Laboratory, Bar Harbor, ME (Cat # 034843). These transgenic mice express the human amyloid precursor protein (APP) gene (isoform 770) containing the Swedish (K670N/M671L), Dutch (E693Q), and Iowa (D694N) mutations under the control of the mouse Thy1 promoter. Mice were housed three to four animals per cage and maintained on a 12-h light/dark cycle (lights out 1800 h) with *ad libitum* access to rodent chow and water in their home cages. Mice weighed 23–28 g at the time of euthanasia. All experiments were approved by the Institutional Animal Care and Use Committees at WCM and were in accordance with the National Institutes of Health Guide for the Care and Use of Laboratory Animals guidelines.

### 2.2 AOF model of perimenopause

Induction of AOF by VCD treatment in mice has been shown to successfully recapitulate the gradual time course of hormonal fluctuations seen from peri- to post-menopause phases in humans (reviewed in Van Kempen et al., 2011; Marques-Lopes et al., 2018). The AOF model can be applied to any mouse genotype and can be used to separate hormonal effects from aging effects (Van Kempen et al., 2014; Brooks et al., 2016; Marques-Lopes et al., 2017). Low dose injections of VCD selectively remove the primary follicles of the ovary and do not negatively affect peripheral tissues or kidney and liver function (Haas et al., 2007; Sahambi et al., 2008; Wright et al., 2008; Brooks et al., 2016). VCD does not increase inflammation markers in the brain including the hippocampus (Van Kempen et al., 2014).

#### 2.2.1 AOF induction

**Figure 1:**
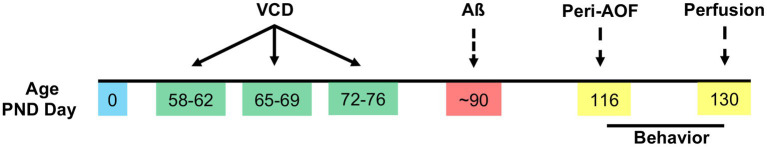
Timeline of experimental procedures. VCD (130 mg/kg, i.p.) was injected for 3 weeks, 5 days per week starting around postnatal day (PND) 58. The deposition of β-amyloid (Aβ) plaques was expected to begin at ~PND 90. Behavioral assessments were performed for 2 weeks following the initiation of the peri-AOF phase. Brains were harvested at ~PND 130.

Gonadally intact 50 to 55-postnatal-day-old female mice were injected with 130 mg/kg VCD (cat. # S453005 Millipore Sigma, St. Louis, MO) in sesame oil (cat. # 8008-74-0 Millipore Sigma) for 5 sequential days per week for 3 weeks (Van Kempen et al., 2011; Marques-Lopes et al., 2017). Control mice were injected with sesame oil only. The time-point corresponding to the peri-AOF stage (58 days after the first VCD injection) was determined in prior studies (Lohff et al., 2005; Haas et al., 2007; Van Kempen et al., 2014). At the peri-AOF phase, the mice are about 3.5 months old and have irregular and extended estrous cycles paralleled by elevated plasma follicle stimulating hormone (Mayer et al., 2004; Lohff et al., 2005; Harsh et al., 2007). Behavioral assessments were initiated at the start of the peri-AOF stage. A timeline of the experimental procedures is presented in Figure 1.

#### 2.2.2 Estrous cycle determination

At the time of euthanasia, vaginal smears (Turner and Bagnara, 1971) were taken to determine estrous cycle stage using cytological examination. Less than 3 mice were in proestrus (i.e., elevated estrogen levels) as euthanasia usually occurred late morning/early afternoon. In addition, part of the peri-AOF model includes the surges of increased estrogen levels seen in proestrus along with the erratic cycle and these animals were included to recapitulate the hormonal fluctuations seen in humans. The majority of oil and VCD treated female mice from both the WT and SWDI genotypes used in this study were in estrus (declining estrogen levels) or diestrus (low estrogen and progesterone levels) on the day of euthanasia.

### 2.3 Antibodies

4G8: A mouse monoclonal antibody raised against amino acid residues 17–24 of beta-amyloid (4G8, Biolegend Cat. # 800701) was used for immunoperoxidase experiments. 4G8 beta-amyloid antibody reacts to abnormally processed isoforms, as well precursor APP forms (manufacturer’s instructions). This antibody labels CAA as well as parenchymal Aβ plaques (Alafuzoff et al., 2009; Kövari et al., 2013; Marazuela et al., 2022), given that diffuse plaques are also seen in SWDI mice (Davis et al., 2004). CD31: A polyclonal goat anti-CD31/PECAM-1 antiserum (Cat. # AF3628, R&D Systems) was used for immunofluorescence experiments to label blood vessels. The antibody detects mouse CD31/PECAM-1 in direct ELISAs and Western blots and mouse CD31 and rat CD 31 in flow cytometry (manufacturer’s datasheet). GFAP: A rabbit polyclonal antibody (Abcam # ab7260; lot # GR20948-21; RRID:AB_305808) raised against a full-length protein corresponding to human GFAP was employed for immunoperoxidase experiments. On Western blot this antibody recognized a 48 kDa and 55 kDa bands corresponding to GFAP (manufacturer’s datasheet). Iba1: A rabbit polyclonal antibody raised against a synthetic peptide corresponding to the C-terminus of Iba1 (#SAR6502; 019–19,741 FUJIFILM Wako Pure Chemical Corporation) was used. The antibody is reactive with rat, mouse and human Iba1 and recognizes a 17 kDA band protein on Western blot (manufacturer’s datasheet).

### 2.4 Brain fixation and histology

At the termination of the behavioral experiments (see below), the brains were processed for immunocytochemistry using established procedures (Milner et al., 2011; Figure 1). For this, mice were deeply anesthetized with sodium pentobarbital (150 mg/kg, i.p.), and their brains perfused with normal saline. Each brain was removed, bisected sagittally, and the right half was placed in 4% paraformaldehyde in 0.1 M phosphate buffer (PB, pH 7.4) for 24 h on a shaker (70 rpm) at 4°C. The forebrain containing the hippocampus was sectioned (40 μm thick) using a vibratome (VT1000X Leica Microsystems, Buffalo Grove, IL) and stored in cryoprotectant (30% sucrose, 30% ethylene glycol in PB) at –20°C until immunocytochemical processing.

For each experiment, two rostral (−2.00 to −2.70 mm from Bregma; Hof et al., 2000) and two caudal (−2.90 to −3.50 mm from Bregma; Hof et al., 2000) hippocampal section from each animal was chosen and then punch coded in the cortex. Tissue sections from one mouse per group then were pooled into single containers (6–8 containers total per experiment) to ensure that sections from each experimental cohort were exposed identically to reagents (Milner et al., 2011).

### 2.5 Light microscopic peroxidase immunocytochemistry

A single cohort of hippocampal sections from each genotype/treatment group was processed for 4G8, Iba1 or GFAP (*N* = 11 per experimental condition). Sections were rinsed in 0.1 M Tris-saline (TS; pH 7.6) followed by an incubation in 0.5% bovine serum albumin (BSA) in TS for 30 min to minimize nonspecific labeling. After, sections were incubated in mouse anti-4G8 (1:4000 dilution), rabbit anti-GFAP (1:6000 dilution) or rabbit anti-Iba-1 (1:4000 dilution) diluted in 0.1% Triton-X and 0.1% BSA in TS for 1-day at room temperature and 1-day at –4°C. Sections were then rinsed in TS and incubated in either goat anti-rabbit IgG conjugated to biotin (GFAP and Iba1; #111–065-144, Jackson ImmunoResearch Inc., West Grove, PA; RRID:AB_2337965) or goat anti-mouse IgG conjugated to biotin (4G8, # 115–065-166, Jackson ImmunoResearch Inc.; RRID:AB_2338569) in 0.1% BSA and TS. Next, sections were washed in TS and incubated with Avidin Biotin Complex (ABC) diluted to half of the manufacturer’s recommended dilution (Vectastain Elite kit, Vector Laboratories, Burlingame, CA) for 30 min. After rinsing in TS, the bound peroxidase was visualized by reaction in 3,3′-diaminobenzidine (Sigma-Aldrich, St. Louis, MO) and 0.003% hydrogen peroxide in TS for 6-min (4G8), 3-min (GFAP), or 8-min (Iba1). All primary and secondary antibody incubations were carried out at 145 rpm whereas all rinses were conducted at 90 rpm on a rotator shaker. Sections were mounted in 0.05 M PB onto gelatin-coated glass slides, dehydrated through an ascending series of alcohol to xylene, and coverslipped with DPX (Sigma-Aldrich).

### 2.6 Image acquisition and field densitometry

Densitometric quantification for 4G8, Iba1 and GFAP labeling within the hippocampus was performed using previously described methods (Williams et al., 2011; Williams and Milner, 2011; Pierce et al., 2014). The analysis was performed by investigators blinded to experimental conditions to ensure unbiased quantification of the data. Sections were photographed with a Nikon Eclipse 80i microscope using a Micropublisher 5.0 digital camera (Q-imaging, BC, Canada) and IP Lab software (Scanalytics IPLab, RRID: SCR_002775). For each antibody, one rostral and one caudal section from each animal were photographed at 4x for the densitometric analysis. Regions of interest (ROI) were first identified in the images (ranging from 50 to 75μm^2^ but the same for each ROI) and then the average pixel density for each ROI was determined using ImageJ64 (Image J, RRID:SCR_003070) software. ROIs within four subregions of the rostral and caudal hippocampus were selected: (1) CA1: stratum oriens (SO), pyramidal cell layer (PCL), stratum radiatum (SR) and stratum lacunosum-moleculare (SLM); (2) CA2/3a: SO, PCL, near and distal SR; (3) CA3b: SO, PCL, stratum lucidum (SLu) and SR; (4) Dentate gyrus (DG): the supragranular blade (SG), the infragranular blade (IFG) and the central hilus (Cen) and (5) Subiculum (caudal section). Pixel density of a small region lacking labeling (i.e., corpus callosum or neuropil) was subtracted from ROI measurements to control for illumination variations between images and to compensate for background labeling. Prior studies (Pierce et al., 2014) have shown a strong linear correlation between average pixel density and actual transmittance demonstrating the accuracy of the technique. High magnification images showing examples of labeling in the figures were taken at 10x.

### 2.7 Dual light microscopic immunolabeling

Selected hippocampal sections were processed for triple labeling of 4G8, CD31, and either GFAP or Iba1. Sections were rinsed in TS followed by an incubation in 0.5% BSA in TS for 30-min. After rinsing in TS, sections were incubated in mouse anti-4G8 (1:4000 dilution), goat anti-CD31, and either rabbit anti-GFAP (1:2000 dilution) or rabbit anti-Iba1 (1:1500 dilution) diluted in 0.25% Triton-X and 0.1% BSA in TS for 1-day at room temperature and 1-day at –4°C. Sections were processed for immunoperoxidase labeling of 4G8 as described above. Next, sections were rinsed in phosphate buffered saline (PBS) and then incubated in AlexaFluor 594 donkey anti-goat IgG (H + L) (Cat.# A-11058; Thermo Fisher; 1:500 dilution) and AlexaFluor 647 donkey anti-rabbit IgG (H + L) (Cat.# A-31573; Thermo Fisher; 1:500 dilution) for 1-h. Sections were rinsed in PBS, mounted on Superfrost plus coated slides (Cat.# 10,135,642, Thermo Fisher) and coverslipped with Prolong Gold Anti-Fade Mountant (Cat # P36930, Thermo Fisher). Immunofluorescent images in X-Y plane were acquired on an Olympus BX61 upright microscope equipped with a Hamamatsu C13440 Orca-Flash 4.0 camera using 594 nm and 647 nm lasers with cellSens imaging software. Vsi files from the upright microscope were exported, converted to TIFF files and processed in ImageJ software. The images were background subtracted to reduce auto-fluorescence. 4G8-labeling was photographed on a Nikon 80i light microscope with a Micropublisher 5.0 digital camera. Brightfield images were scaled to size and anatomical structure, and superimposed with fluorescent images in pseudo-colors using ImageJ.

### 2.8 Behavioral assessments

Mice were assessed sequentially over 2 weeks on the Novel Object Recognition, Y maze and Barnes maze tests as described in prior studies (Park et al., 2008, 2020; Figure 1). The behavioral tests were performed by the same investigator at the same time each day. Results were recorded using ANY-maze (Stoelting Co., Wood dale, IL). Mice were habituated to the room for 2-h per day for 5 days before starting behavioral tests. On training and testing days, mice were acclimated to the room for 1-h prior to the beginning of each session. The order of the Novel Object and Y-maze tests were counterbalanced; mice were rested 1-day between these tests. Behavioral apparatuses were cleaned with 70% ethanol between trials.

#### 2.8.1 Novel object recognition

The apparatus (height 30 cm × width 28 cm × length 46 cm) consisted of an open field chamber with dim illumination throughout. Day 1 (habituation phase): each mouse was placed in the center arena without any objects and allowed to explore for 5 min. Day 2 (familiarization phase): Two identical objects (type A) were placed on the floor of the area and the mouse was allowed to explore for 5 min. Mice then rested for 30-min. Day 2 (exploration phase): One of the type A objects was replaced with a novel object (type B) and the mouse was allowed to explore for 5 min. For each phase, the total distance traveled, the average speed, the total object exploration time, and the time spent exploring each one of the two objects was recorded. In day 2 (exploration phase), the discrimination index as percentage time spent exploring the novel object out of the total object exploratory time was calculated for each mouse was calculated.

#### 2.8.2 Y-maze

Spatial working memory was assessed using the Y-maze as described previously (Toyama et al., 2014). The maze consisted of 3 arms (40 cm long, 9.5 cm high, and 4 cm wide, labeled A, B, or C) diverging at a 120° from the central point. Each mouse was placed into one of the arms of the maze (start arm) and allowed to explore only two of the arms for 5 min (training trial). The third arm, which remains closed, was randomly chosen in each trial. After a 30-min inter-trial interval, the closed arm, serving as the novel arm, was opened in the test trial and the mice were returned to the same start arm and were allowed to explore all three arms for.

5-min (test trial). The sequence of arm entries was manually recorded. A mouse was considered to have entered an arm when all 4 paws were positioned in the arm runway. An alternation was defined as entries into all the 3 arms on consecutive occasions (e.g., in the sequence ABCBCBCA 2 alternations are counted with the first consecutive ABC and the last consecutive BCA out of 6 consecutive occasions). The maximum possible alternation per each mouse was calculated by measuring the total number of arm entries minus 2. Spontaneous arm alternation was calculated as percentage change using a formula [(number of alternations/maximum possible alternations) × 100] for each mouse. The total number of arms entered during the sessions, which reflect locomotor activity, also was recorded.

#### 2.8.3 Barnes maze

The mice were trained in the apparatus for 4 sequential days. Each training day consisted of 4 trials (with inter-trial intervals of 15 min) of the following sequence: (1) Adaption period. The mouse was placed in the middle of the maze in a 10 cm high cylindrical white start chamber, and the buzzer switched on for 10 s. The mouse was guided with a glass 2-L beaker into the escape hole for 15–20-s. The buzzer was turned off and the mouse was allowed to stay in the escape box for 2-min. (2) Spatial acquisition period. The mouse was placed in the middle of the maze in a 10 cm high cylindrical white start chamber. The video record button was started, and the buzzer was switched on. After 10 s, the chamber was removed, and the mouse was allowed to move around the maze to find an escape hole (maximum 3 min). Immediately after the mouse entered the escape hole, the buzzer was turned off and the mouse was allowed to stay in the tunnel for 1 min. Twenty-four hours after the last training session, mice underwent the probe trial. For this, the mouse was placed in the maze in the white chamber and the buzzer was switched on. After 10 s, the chamber was removed, and the mouse behavior was recorded for 90 s. For each mouse, the latency time, errors, and total length traveled was recorded.

### 2.9 Image adjustments

Images first were adjusted for contrast and sharpness in Adobe Photoshop 9.0 (Adobe Photoshop, RRID:SCR_014199). Next, images were imported into Microsoft PowerPoint 2010, where final adjustments to brightness, sharpness and contrast were achieved. Adjustments were made to the entire image, none of which significantly altered the appearance of the initial raw image.

### 2.10 Data analysis

Data are expressed as means ± SEM. Statistical analyses were conducted using JMP 12 Pro software (JMP, RRID:SCR_014242) or Prism 9 software (Graphpad Prism, RRID:SCR_002798) and significance was set to an alpha <0.05. Differences between groups were compared by two- or three-way analysis of variance (ANOVA) followed by Tukey or Sidak’s post-hoc multiple comparison tests. Differences between 2 groups were determined using student’s t-tests. Graphs were generated using Prism 9 software (Graphpad Prism, RRID:SCR_002798).

## 3 Results

### 3.1 Peri-AOF is associated with increased amyloid fibrils throughout the dentate gyrus and in select regions of the CA1 of SWDI mice

Small vessel disease and dementia are more prevalent in women and may emerge at the onset of menopause. There is, however, little evidence that levels of Aβ are influenced in the hippocampus during perimenopause. To evaluate whether Aβ is increased in the hippocampus of females at a stage of early ovarian failure, female wild type and SWDI mice were administered VCD, or sesame oil as a control, and Aβ was quantified at a stage of AOF compatible to human perimenopause (i.e., peri-AOF). Prior reports of sex differences in Aβ deposition in functionally distinct regions of the hippocampus of SWDI mice (Setti et al., 2022) point to actions of gonadal hormone signaling in amyloidosis, therefore analysis was performed across major regions of the hippocampus.

**Figure 2:**
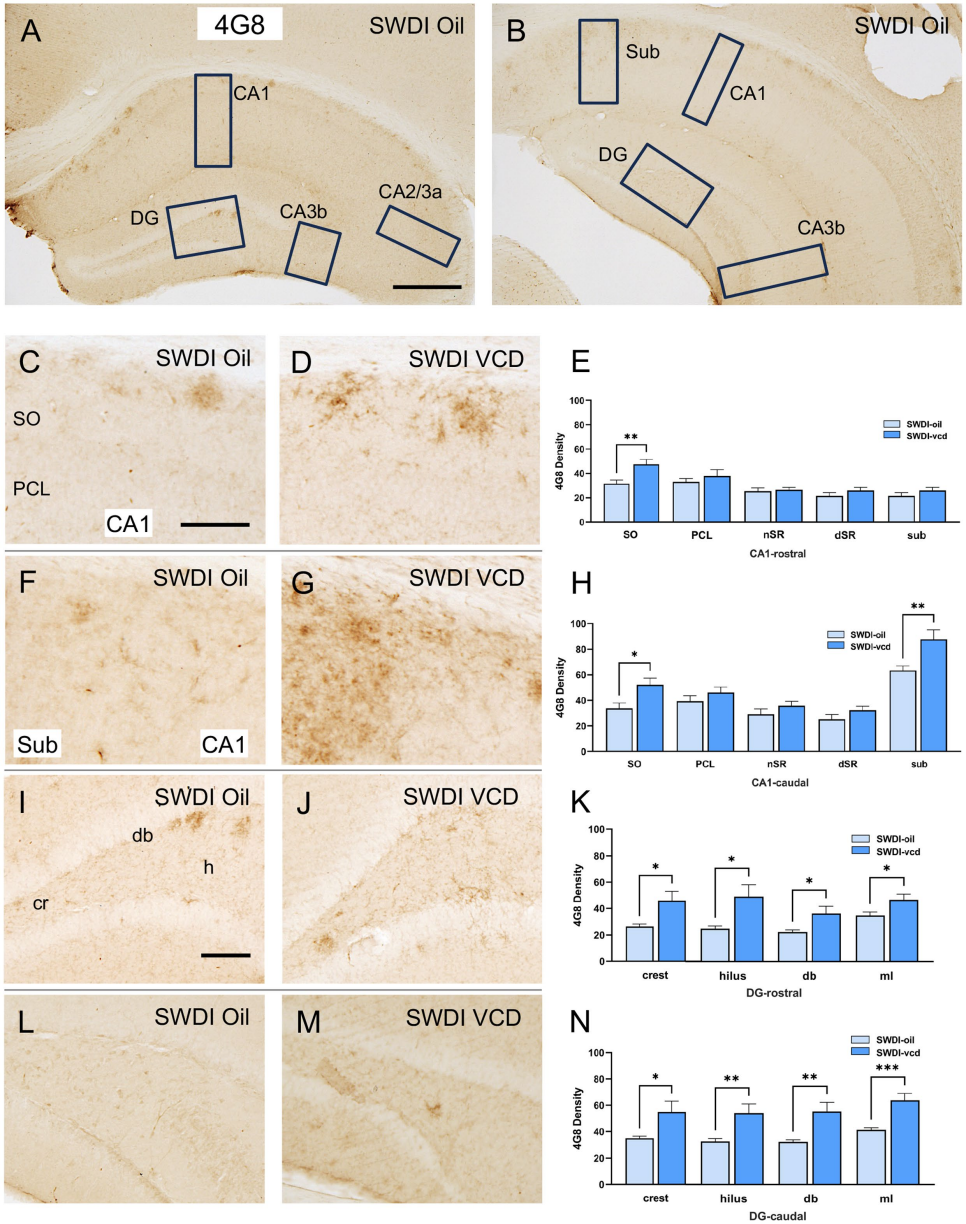
4G8 labeling is increased in select regions of the CA1, subiculum and dentate gyrus of peri-AOF SWDI mice. **(A,B)** Low-magnification photomicrographs of 4G8 labeling in the rostral **(A)** and caudal **(B)** hippocampus. Boxes indicate regions of the CA1, CA3a, CA3b, dentate gyrus (DG) and subiculum (Sub) that were sampled. **(C,D)** Representative photomicrographs showing 4G8 labeling in the rostral CA1 of SWDI-oil **(C)** and SWDI-VCD mice **(D)**. **(E)** In the rostral CA1 SO, SWDI-VCD mice show significantly more 4G8 labeling than compared to WT-VCD mice. **(F,G)** Representative photomicrographs showing 4G8 labeling in the caudal CA1 of SWDI-oil **(F)** and SWDI-VCD **(G)** mice. **(H)** In the caudal CA1 SO and subiculum, SWDI-VCD mice show significantly more 4G8 labeling compared to WT-VCD mice. **(I,J)** Representative photomicrographs showing 4G8 labeling in the rostral DG of SWDI-oil **(I)** and SWDI-VCD mice **(J)** mice. **(K)** In the DG, SWDI-VCD mice compared to SWDI-oil mice show significantly more 4G8 labeling in all subregions of rostral subregions. **(L,M)** Representative photomicrographs showing 4G8 labeling in caudal DG of SWDI-oil **(L)** and SWDI-VCD **(M)** mice. **(N)** In the DG, SWDI-VCD mice compared to SWDI-oil mice show significantly more 4G8 labeling in all caudal subregions. Bars **(A,B)** = 200 μm, **(C,D,F,G,I,L,M)** = 50 μm; *p < 0.05; **p < 0.01; ***p < 0.001 by Student’s paired *t*-test. Data are mean ± SEM, *N* = 11 animals per experimental group.

The density of Aβ was examined in CA1, CA3, DG and subicular subregions within rostral and caudal hippocampus (Figures 2A,B). When comparing labeling separately in rostral and caudal regions, a greater increase in 4G8-labeling in VCD vs. oil SWDI mice was seen in the caudal region [oil = 13 ± 2, VCD = 45 ± 2; *t*_(20)_ = 4.1, *p* < 0.001], with a much smaller increase occurring rostrally [oil = 17 ± 1, VCD = 25 ± 1; *t*_(20)_ = 2.5, *p* < 0.05]. Additionally, as described below, 4G8 labeling was altered across the hippocampus with sublayer specificity.

#### 3.1.1 CA1 and subiculum

Significantly more 4G8 labeling was seen in the rostral CA1 SO [*t*_(20)_ = 3.2, *p* < 0.01] when comparing SWDI-VCD to SWDI-oil mice. Additionally, SWDI-VCD mice also showed higher 4G8 labeling in the caudal CA1 SO compared to SWDI-oil animals [*t*_(20)_ = 2.8, *p* < 0.05]. Unlike the rostral region, it was also shown that SDWI-VCD mice had higher levels of 4G8 labeling in the caudal subiculum [*t*_(20)_ = 3, *p* < 0.01] compared to SWDI-oil mice (Figures 2C–H).

#### 3.1.2 CA3

There was no effect of VCD treatment on the density of 4G8 labeling in any of the subregions of the CA2/CA3A or CA3b in either the rostral or caudal hippocampus (data not shown).

#### 3.1.3 DG

In SWDI-VCD compared to SWDI-oil mice, significantly more 4G8 labeling was seen in all subregions of the rostral and caudal DG. In the rostral region these include the crest [*t*_(20)_ = 2.7, *p* < 0.05], hilus [*t*_(20)_ = 2.7, *p* < 0.05], dorsal blade [db; *t*_(20)_ = 2.4, *p* < 0.05], and molecular layer (ml; *t*_(20)_ = 2.5, *p* < 0.05). Caudally, the crest (*t*_(20)_ = 2.4, *p* < 0.05), hilus (t_(20)_ = 3.0, *p* < 0.01), db [*t*_(20)_ = 3.3, *p* < 0.01], and ml [*t*_(20)_ = 4.0, *p* < 0.001] also showed higher 4G8 labeling (Figures 2I–N).

### 3.2 Peri-AOF is associated with increased levels of reactive astrocytes in select regions of the caudal CA1 of SWDI mice

Astrocytes are a critical component of the neurovascular unit and play key roles in coordinating neurovascular signaling through inflammatory, metabolic, and trophic processes (Li et al., 2023). They also aid in the removal of Aβ from the brain parenchyma by mediating amyloid efflux into the cerebral vasculature (Liu et al., 2018). Astrocyte function is impacted by sex, partly through the actions of estrogen (Santos-Galindo et al., 2011; Lu et al., 2020; Chowen and Garcia-Segura, 2021). Thus, astrocytes would be expected to play an important role in cerebrovascular amyloidosis during ovarian failure, but there is little evidence that astrocyte activity is altered across hippocampal subregions of intact or reproductively compromised females.

**Figure 3:**
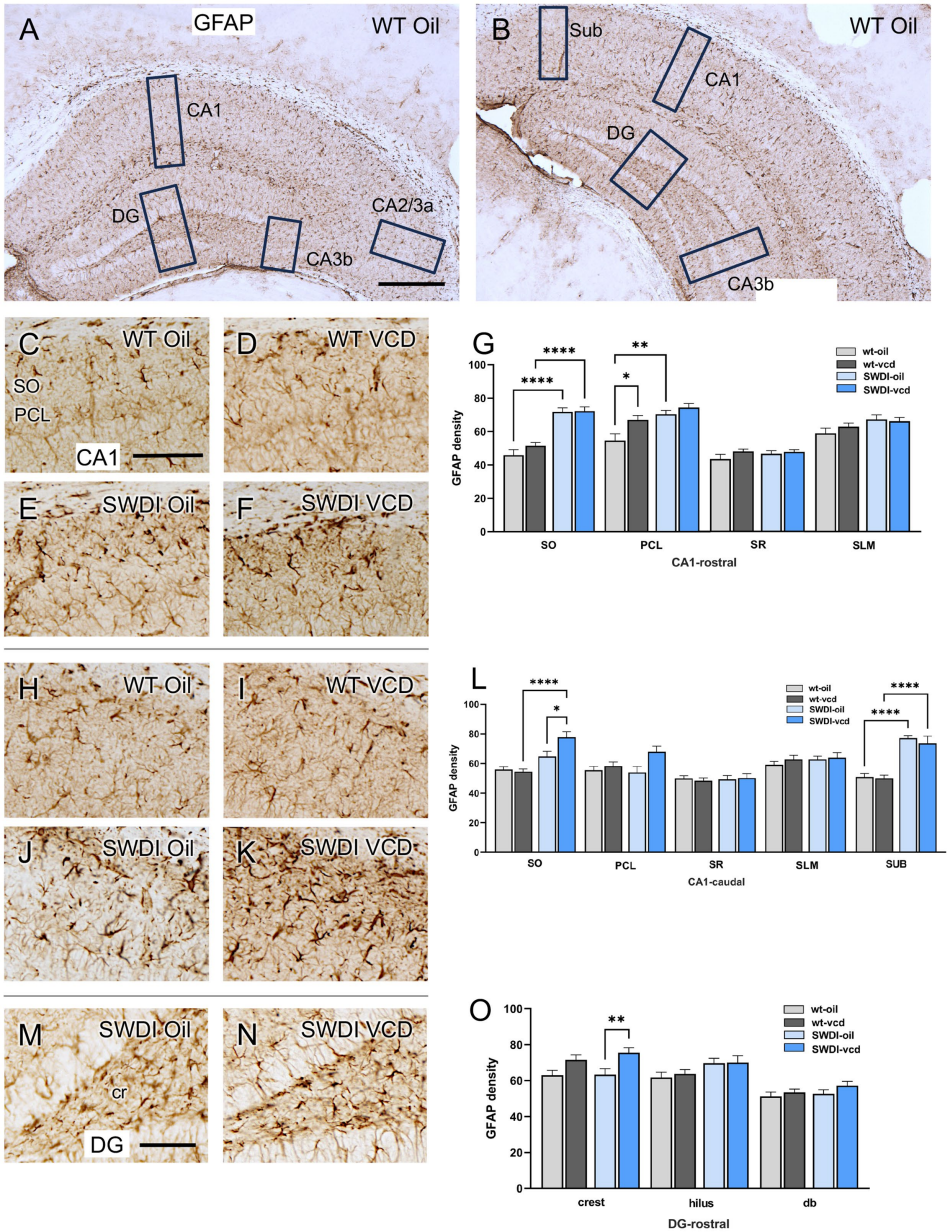
Increased GFAP labeling is associated with peri-AOF and/or SWDI genotype in select regions of the hippocampus. **(A,B)** Low magnification photomicrographs of GFAP labeling in the rostral **(A)** and caudal **(B)** hippocampus. Boxes indicate regions of the CA1, CA3a, CA3b, dentate gyrus (DG) and subiculum (Sub) that were sampled. **(C–F)** Representative micrographs showing GFAP labeling in the rostral CA1 of WT-Oil **(C)**, WT-VCD **(D)**, SWDI-Oil **(E)**, and SWDI-VCD **(F)** mice. **(G)** In the rostral CA1, the density of GFAP labeling was increased in the SO of SWDI mice compared to WT mice, irrespective of AOF treatment. In the PCL, GFAP was increased in both WT-VCD and SWDI-oil mice compared to WT-oil mice. **(H–K)** Representative micrographs showing GFAP labeling in the caudal CA1/subiculum of WT-Oil **(H)**, WT-VCD **(I)**, SWDI-Oil **(J)**, and SWDI-VCD **(K)** mice. **(L)** In caudal CA1 SO, the density of GFAP labeling is increased in SWDI-VCD mice compared to WT-VCD mice and SWDI-oil mice. In caudal subiculum, the density of GFAP labeling is increased in SWDI-WT and SWDI-VCD mice compared to their WT counterparts. **(M,N)** Representative micrographs showing GFAP-labeling in the rostral DG of SWDI-Oil **(M)**, and SWDI-VCD **(N)** mice. **(O)** SWDI-VCD mice show significantly more GFAP labeling in rostral DG crest compared to SWDI-oil mice. Bars **(A,B)** = 200 μm; **(C–F,H–K,M,N)** = 50 μm; *p < 0.05; **p < 0.01; ****p < 0.0001 by two-way ANOVA with Tukey’s or Sidak’s *post hoc* multiple comparison analysis. Data are mean ± SEM, *N* = 11 animals per experimental group.

The density of the astrocytic marker GFAP was examined in CA1, CA3, DG and subicular subregions within rostral and caudal hippocampus (Figures 3A,B) of oil and VCD-treated SWDI and WT mice. Consistent with our prior studies in female mouse brain (Milner et al., 2022), GFAP-labeled cells were found throughout the CA1, CA3 and DG (Figure 3). Representative micrographs of GFAP-labeling are shown for rostral CA1 (Figures 3C–F), caudal CA1/subiculum (Figures 3H–K) and the dentate gyrus (Figures 3M,N).

#### 3.2.1 CA1 and subiculum

In the rostral CA1 there was a main effect of treatment in the PCL region (*F*_treatment_ = 8.02, *p* < 0.0072). Post-hoc analysis showed that the density of GFAP labeling in PCL was greater (*p* < 0.05) in WT’s treated with VCD compared to oil (Figure 3G). There was also a main effect of genotype in the SO (*F*_genotype_ = 80.62, *p* < 0.0001) and PCL of the rostral CA1 (*F*_genotype_ = 16.07, *p* < 0.0003), with SWDI mice showing significantly higher GFAP labeling. In the remainder of rostral CA1 subregions, neither VCD treatment nor genotype affected the density of GFAP.

In caudal CA1, the density of GFAP labeling was increased in the SO region of the CA1 and subiculum in SWDI compared to WT mice (Figure 3L). Notably, only in the caudal CA1 SO subregion was there a significant interaction (*F*_interaction_ = 6.3, *p* < 0.016) of treatment and genotype in the density of GFAP-labeling, post-hoc analysis showing that SWDI-VCD mice (Figure 3L) had significantly greater GFAP density compared to SWDI-Oil mice (*p* < 0.05). In contrast, in the subiculum of the caudal CA1 there was an effect of genotype (*F*_genotype_ = 65, *p* < 0.0001) with significantly greater density of GFAP labeling in SWDI mice compared to WT mice (*p* < 0.0001).

#### 3.2.2 CA3

There was no effect of treatment or genotype on the density of GFAP in any of the subregions of the CA2/CA3A or CA3b (data not shown).

#### 3.2.3 DG

There was a significant effect of treatment (*F*_treatment_ = 12, *p* < 0.001) on GFAP labeling in the crest of the rostral DG of the SWDI mice. *Post hoc* testing showed that SWDI-VCD compared to SWDI-Oil mice had a significantly greater GFAP density (*p* < 0.01) in the crest of the hilus (Figure 3O). There were no effects of treatment in the WT mice on GFAP density in the rostral DG. Moreover, there were no significant effects of treatment or genotype on GFAP density in the caudal DG (data not shown).

These patterns of GFAP labeling suggest that early-stage CAA is associated with increased astrocyte activity in select hippocampal areas of the rostro-caudal CA1 and caudal SUB. Significantly, only the caudal CA1 SO showed further signs of astrocyte reactivity when early cerebrovascular amyloidosis was coupled to AOF.

### 3.3 Astrocytes in AOF SWDI mice are associated with amyloid pathology

Mounting evidence supports that reactive glia are associated with aggregated Aβ in the hippocampus and cortex of AD animal models and human brain samples (Bandyopadhyay, 2021). In the SWDI model of CAA, early onset accumulation of Aβ protein is found in diffuse parenchymal plaques, cerebral microvessels and is associated with vascular degeneration and neuroinflammation especially in the subiculum (Miao et al., 2005a,b). In this model, abundant neuroinflammatory reactive astrocytes and activated microglia strongly associate with the cerebral microvascular fibrillar Aβ deposits.

**Figure 4:**
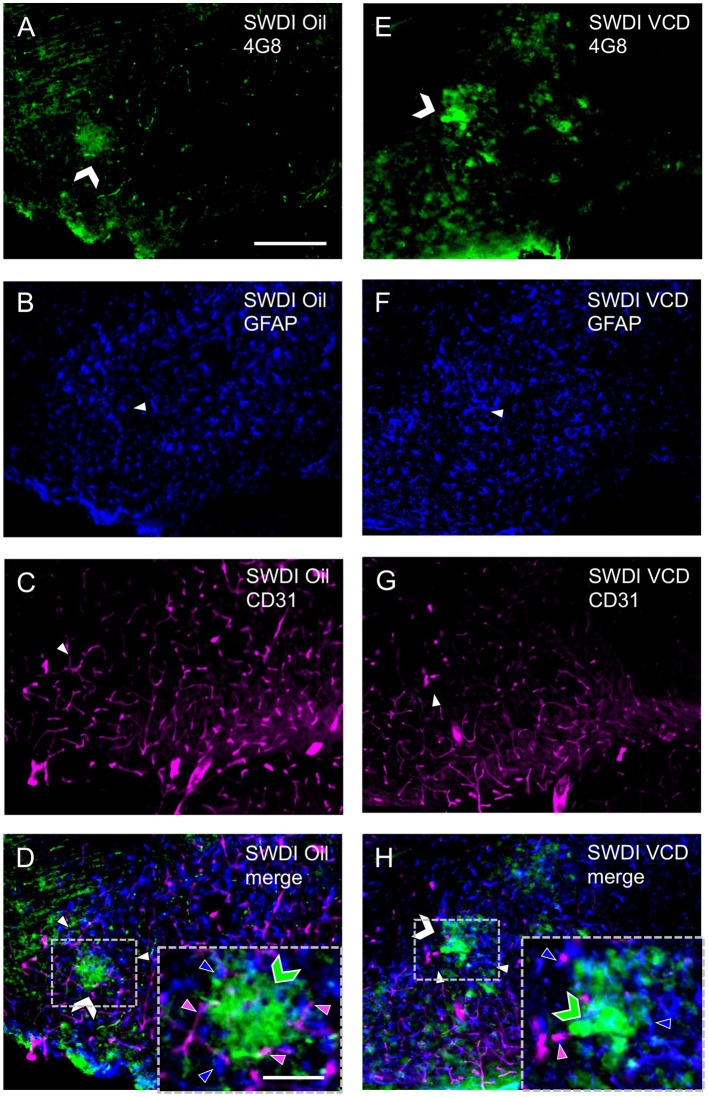
Qualitative analysis shows Aβ, astrocytes and blood vessels are located in spatial proximity in the caudal subiculum of SWDI mice. **(A–D)** Section from SWDI oil-treated mouse shows labeling of 4G8 **(A)**, GFAP **(B)**, and vascular marker CD31 **(C)**. A merged image **(D)** shows overlapping labeling for 4G8, GFAP and CD31. As shown in the inset (area in dashed box) labeling for 4G8 (green chevron) is seen in proximity to labeling for astrocyte (blue arrow head) and vascular (magenta arrow head) markers. **(E–H)** Section from SWDI VCD-treated mouse shows labeling for 4G8 **(E)**, GFAP **(F)**, and CD31 **(G)**. A merged image shows overlap of 4G8, GFAP and CD31 **(H)**, which can be seen at a higher magnification in the area bounded by the dashed box (inset). Bars **(A–H)** = 200 μm. Bar **(D,H)** = 75 μm.

Similar to prior studies in SWDI mice (Xu et al., 2007), qualitative analysis showed that GFAP-labeling (Figures 4A,E) and 4G8-labeling (Figures 4B,F) were found in close proximity (Figures 4D,H) in the dorsal subiculum of both Oil and VCD injected mice. The GFAP-labeled astrocytes mostly surround clusters of 4G8-labeling, especially in the VCD SWDI mice (Figures 4D,H). Moreover, blood vessels, identified by CD31 immunolabeling (Figures 4C,G), were closely associated with GFAP-labeled cells in the parenchyma but few blood vessels overlapped regions with dense GFAP and 4G8-labeling (Figures 4D,H). This could indicate an increase in vascular amyloid burden as shown in the data presented in Figure 2 and suggest a decrease in cerebral vascular densities in the hippocampus as previously reported (Miao et al., 2005a,b; Xu et al., 2007; Robison et al., 2019; Maniskas et al., 2021).

### 3.4 Increased microglia activation in select regions of the CA1 of SWDI mice

Microglia are resident brain macrophages implicated in regulation of neuroinflammatory states and cognitive function (Cornell et al., 2022). The protein Iba1 is constitutively expressed in microglia and upregulated when they enter an activated stage (Imai et al., 1996; Sasaki et al., 2001), as is commonly reported in the context of aging and neurodegenerative disorders (Prinz et al., 2021). Significantly, microglia activity is associated with ovarian hormone changes. For example, ovariectomy results in an increase in Iba1 in middle-aged mice (Sárvári et al., 2017), and signs of microglia reactivity are decreased by estradiol in hippocampus of aged ovariectomized animals (Lei et al., 2003). In addition, ovariectomy increases the labeling of macrophage antigen complex-1, another marker of reactive microglia, in the hippocampus of aged mice (Benedusi et al., 2012). In the context of neurodegenerative disease in females, gonadal hormones further impact microglia, as chronic estrogen deficiency is associated with increased microglial activation and neurodegeneration in an AD mouse model (Prat et al., 2011). Expression of Iba1 in the hippocampus might be expected to be affected by ovarian failure during cerebrovascular amyloidosis, however, there is little evidence for this.

**Figure 5:**
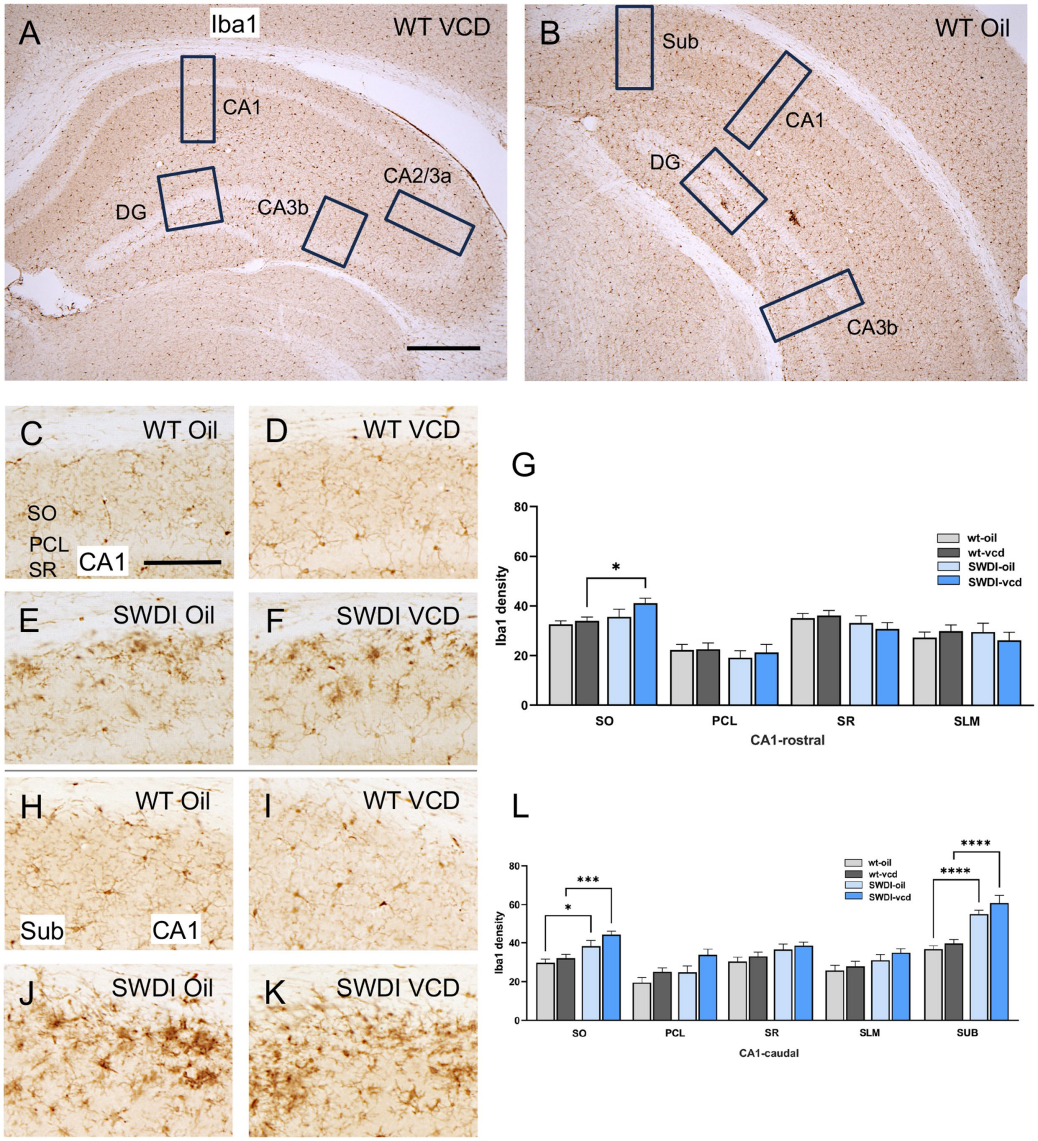
Iba1 labeling is elevated in select hippocampal regions of SWDI mice. **(A,B)** Low magnification photomicrographs of Iba1 labeling the rostral **(A)** and caudal **(B)** hippocampus. Boxes indicate regions of the CA1, CA3a, CA3b, dentate gyrus (DG) and subiculum (Sub) that were sampled. **(C–F)** Representative micrographs showing Iba1 labeling in the rostral CA1 of WT-Oil **(C)**, WT-VCD **(D)**, SWDI-Oil. **(E)**, and SWDI-VCD **(F)** mice. **(G)** The density of Iba1 labeling in rostral CA1 SO is increased in SWDI-VCD mice compared to WT-VCD mice. **(H–K)** Representative micrographs showing Iba1 labeling in the caudal CA1/subiculum of WT-Oil **(H)**, WT-VCD **(I)**, SWDI-Oil **(J)**, and SWDI-VCD **(K)** mice. **(L)** The density of Iba1 labeling in caudal CA1 SO and subiculum is increased in SWDI-WT and SWDI-VCD mice compared to their WT counterparts. Bars **(A,B)** = 200 μm, **(C–F,H–K)** = 50 μm; *p < 0.05; ***p < 0.001; ****p < 0.0001 by two-way ANOVA with Tukey’s or Sidak’s *post hoc* multiple comparison analysis. Data are mean ± SEM, *N* = 10–11 animals per experimental group.

The density of Iba1 was examined in CA1, CA3, DG and subiculum subregions within the rostral and caudal hippocampus (Figures 5A,B). As shown in an example from a WT female, Iba1-labeled cells were found scattered throughout all lamina in the CA1, CA3 and DG; however, the pyramidal and granule cell layers contain fewer Iba1-labeled cells (Figure 5A). Representative micrographs showing the distribution of Iba1 labeling in the rostral and caudal CA1 from each of the four animal groups are shown in Figures 5C–F,H respectively.

#### 3.4.1 CA1 and subiculum

In the rostral CA1 there were no significant differences in the density of Iba1 between WT-oil and WT-VCD mice in any subregion of the rostral or caudal CA1, or in the subiculum (Figures 5G,L). However, there was a genotype effect in the SO of the caudal CA1 (*F*_genotype_ = 21, *p* < 0.001) and in the subiculum (*F*_genotype_ = 55, *p* < 0.0001). In both the CA1 SO (*p* < 0.05) and subiculum (*p* < 0.0001), SWDI-oil compared WT-oil had greater densities of Iba1 labeling (Figure 5L). SWDI-VCD compared to WT-VCD mice had significantly greater densities of Iba1 labeling in rostral and caudal CA1 SO (*p < 0.05) and in the subiculum (*p* < 0.0001).

#### 3.4.2 CA3 and DG

There was no effect of treatment or genotype on the density of Iba1 labeling in any of the subregions of the CA2/CA3A or CA3b or DG (data not shown).

**Figure 6:**
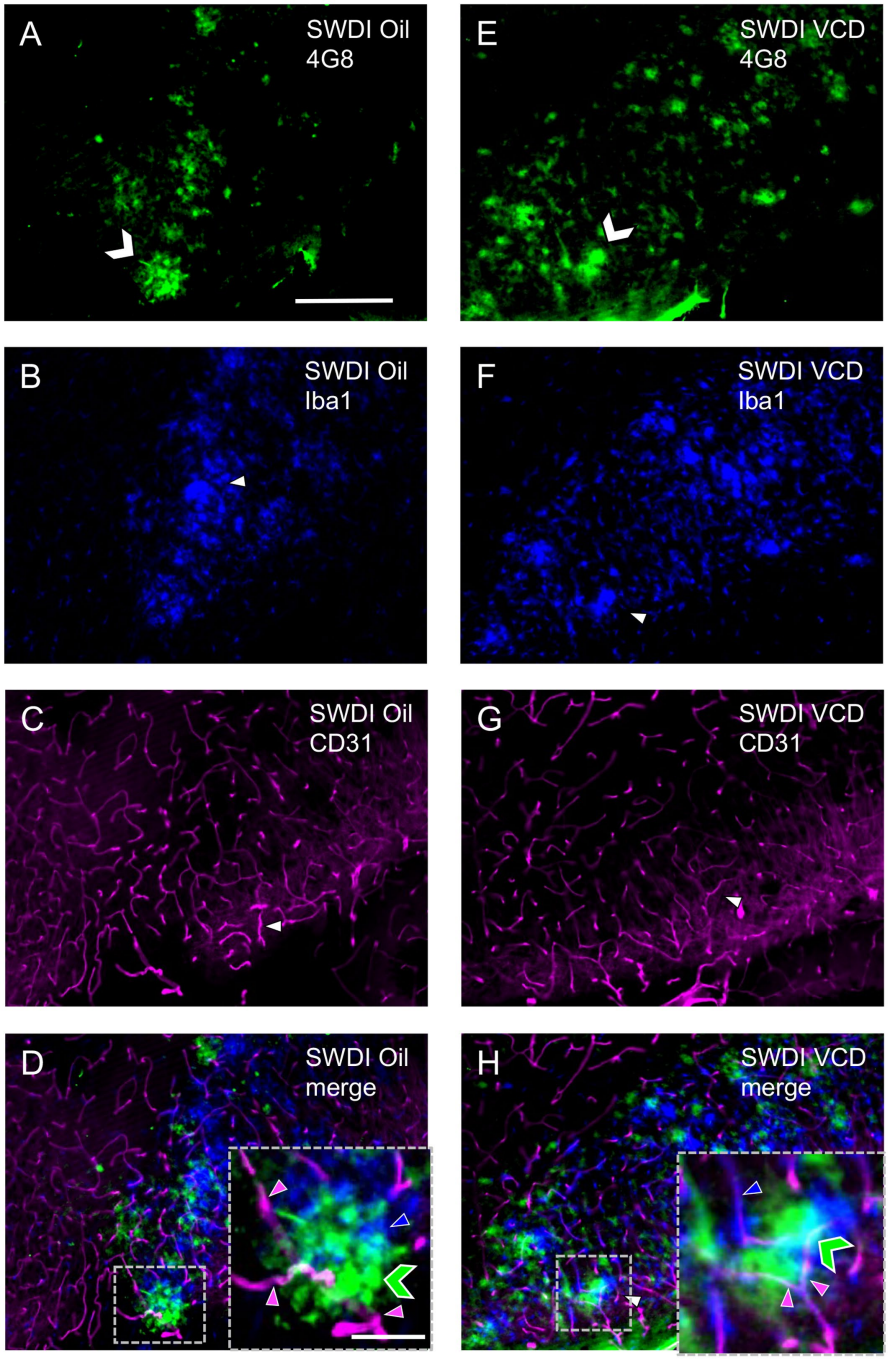
Qualitative analysis shows overlapping labeling for 4G8, Iba1 and CD31 in the caudal subiculum of SWDI mice. **(A–D)** Section from SWDI oil-treated mouse shows labeling for 4G8 **(A)**, Iba1 **(B)**, and CD31 **(C)**. A merged image **(D)** shows overlapping areas of 4G8 (green chevron), Iba1 (blue arrowhead) and CD31 (magenta arrowhead) labeling at low and high magnification as shown in the area bounded by the dashed box (inset). **(E–H)** Section from SWDI VCD-treated mouse shows labeling of 4G8 **(E)**, Iba1 **(F)**, and CD31 **(G)**. A merged image **(H)** showing 4G8, Iba1 and CD31 labeling in close spatial proximity is shown at low and high magnification in the area contained in the dashed box (inset). Bars **(A–H)** = 200 μm. Bar **(D,H)** = 75 μm.

Similar to GFAP, qualitative analysis showed that Iba1 labeling overlapped with 4G8-labeled fibrils in the dorsal subiculum of both Oil (Figures 6A–D) and VCD (Figures 6E–H) injected mice. The Iba1-labeled microglia mostly surround clusters of 4G8-labeling, especially in the VCD-injected SWDI mice (Figures 6D,H). Moreover, few blood vessels were associated with Iba1-labeled microglia and 4G8-labeling (Figures 6D,H).

These results of Iba1 labeling suggest that the SWDI genotype at an early stage of cerebral Aβ expression influences microglia activation in the caudal CA1 SO and the SUB. However, in contrast to GFAP, there is no further impact of peri-AOF on microglia activation at a similar stage of amyloid progression. These results, in concert with the finding of increased GFAP in select hippocampal regions, suggest that there is a dissociation in activation states across astrocytes and microglia in response to peri-AOF-associated cerebrovascular Aβ expression.

### 3.5 Cognitive impairment in female SWDI mice independent of VCD treatment

Neuroinflammation is intrinsically linked with dementia and the progression of cognitive impairment (Heneka et al., 2015), however the impact of early ovarian failure on cognitive function in mice with CAA is unknown. To assess the behavioral consequences of early ovarian decline and cerebrovascular amyloidosis, SWDI mice at peri-AOF were tested in different cognitive tasks (Y-maze alternation test, novel object recognition, and spatial navigation using the Barnes maze).

#### 3.5.1 Y-maze

**Figure 7:**
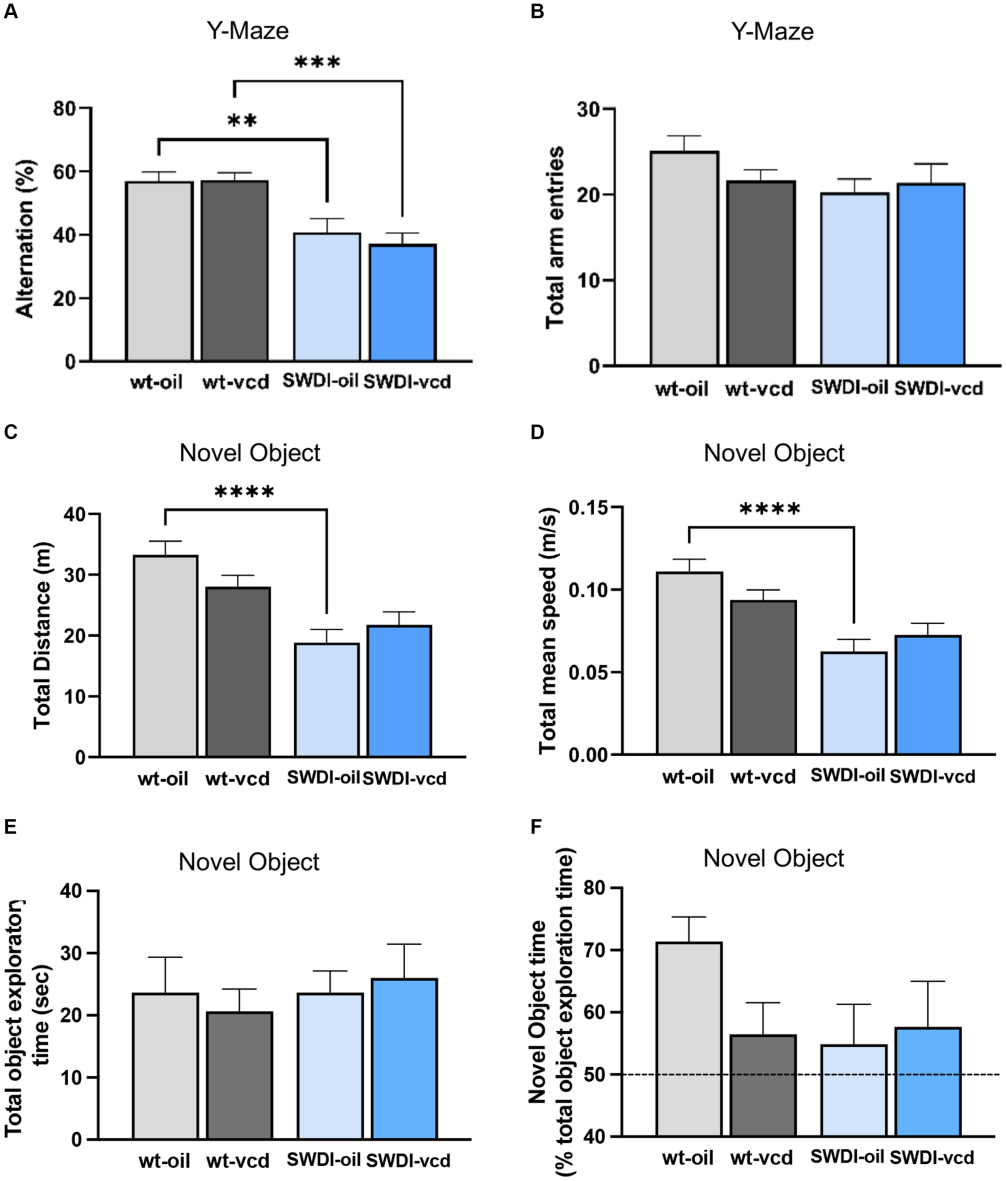
Cognition is impaired in the Y-maze, but not in the novel object recognition test, in SWDI compared to WT mice. **(A,B)** In the Y-maze test, arm alternation behavior was reduced in SWDI mice compared to WT mice with no differences in number of arm entries. **(C,D)** Spontaneous motor activity was reduced in SWDI compared to WT mice as observed in the novel object recognition test (**p < 0.01; ***p < 0.001; ****p < 0.0001) by two-way ANOVA with Sidak’s *post hoc* multiple comparison analysis. No significant differences were observed between groups in the total object exploratory time and percentage time spent exploring the novel object. Data are mean ± SEM, *N* = 11 animals per experimental group.

A genotype effect was observed in the arm alternation behavior, indicative of willingness to explore new environments (*F*_genotype_ = 29, *p* < 0.0001). Both oil and VCD treated SWDI mice had a significantly lower alternation percentage compared to oil and VCD treated WT mice (*p* < 0.01, *p* < 0.001 respectively) with no change in total arm entries (Figures 7A,B).

#### 3.5.2 Novel object

A genotype effect was seen in locomotor activity as assessed by total distance (*F*_genotype_ = 24.1, *p* < 0.0001) and total mean speed (*F*_genotype_ = 24.6, *p* < 0.0001). SWDI-Oil compared to WT-Oil mice had lower total distance (*p* < 0.0001) and lower mean speed (*p* < 0.0001) (Figures 7C,D). No significant differences across groups were observed in the total time spent exploring the two objects (Figure 7E). Also, no genotype or treatment effect was observed in the percent time of novel object exploration (Figure 7F); however, a trend toward a decrease in novel object exploration time was seen in WT-VCD, SWDI-Oil, and SWDI-VCD compared to WT-OIL controls.

#### 3.5.3 Barnes maze

**Figure 8:**
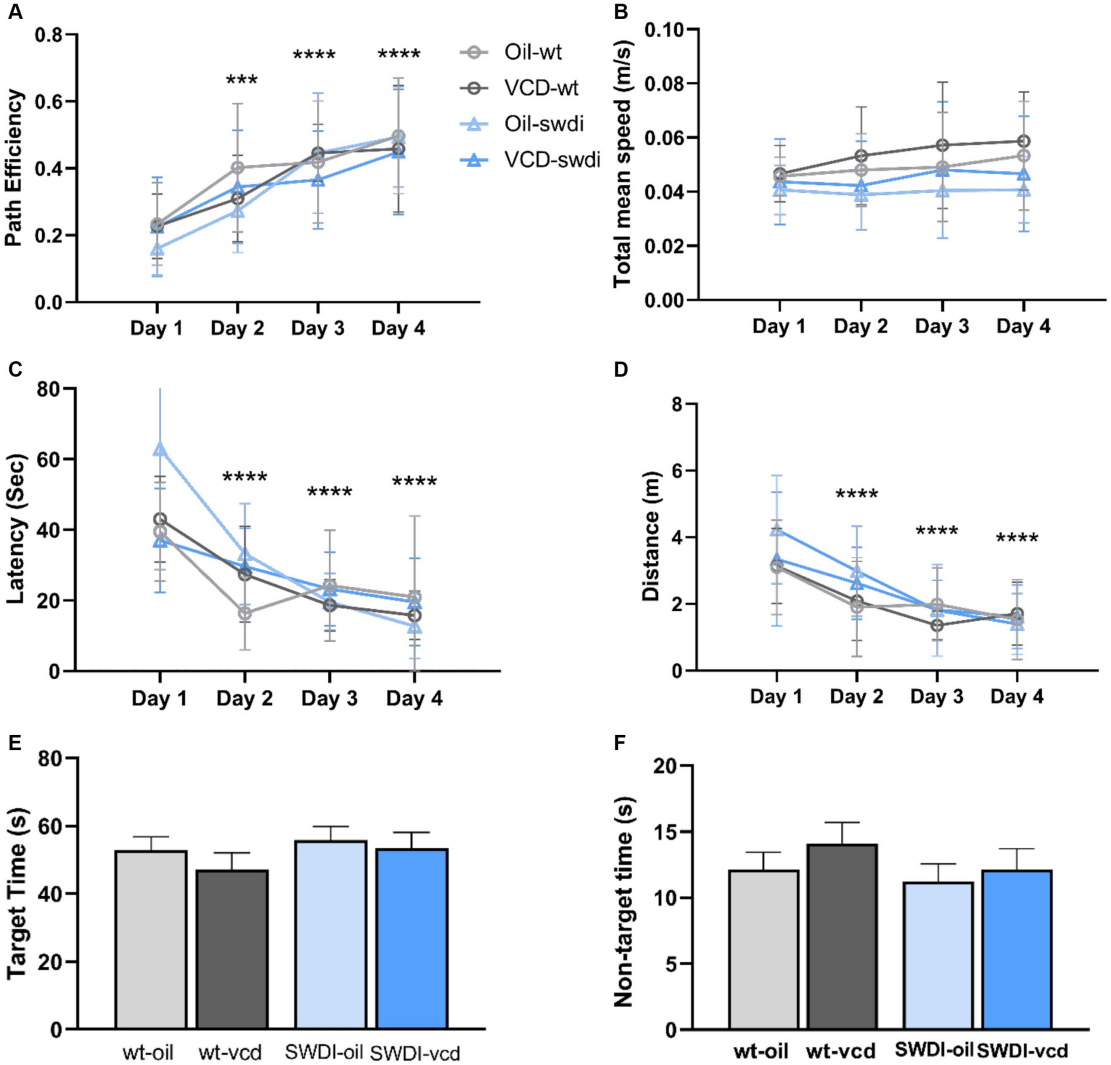
There were no differences in cognition performance in the Barnes maze due to genotype or VCD treatment. **(A–F)** Differences in learning and memory **(A,C,E,F)** or motor activity **(B,D)** were not observed in the Barnes maze in SWDI mice compared to WT mice (***p < 0.001; ****p < 0.0001) by three-way ANOVA with Dunnett’s *post hoc* multiple comparison analysis. Data are mean ± SEM, *N* = 11 animals per experimental group.

There were no significant differences in cognitive performance or motor activity according to either genotype or treatment between (Figures 8A–D). When tested 24- h after the last acquisition training session, no differences were found in time spent in the target or non-target quadrants in the probe trial (Figures 8E,F).

These data demonstrate that female SWDI mice display impaired performance in select cognitive tasks, as has been reported previously (Xu et al., 2014; Setti et al., 2022). However, early ovarian failure does not exacerbate these behavioral deficits. Together with the anatomical results, our data show that early menopause alters hippocampal Aβ deposition and activation of astrocytes with sublayer specificity in SWDI mice compared to wildtype animals (Figures 2, 3) but does not aggravate cognitive deficits.

## 4 Discussion

Women may bear a disproportionate burden of both small vessel disease (Patel et al., 2020), and dementia (Beam et al., 2018), however, the biological processes contributing to dementia-related neuropathology and cerebrovascular dysfunction are unclear. Given that the risk for dementia may emerge as women transition to menopause, we explored the role of early ovarian failure on markers of neuroinflammation in the hippocampus, a dementia-sensitive brain area, using a model of cerebrovascular angiopathy, a significant contributor to dementia. To further characterize the role of ovarian decline on the progression of amyloidosis-associated neuroinflammation, conditions were optimized in young mice to capture the earliest stages of ovarian failure and amyloidosis, thus identifying potential early disease markers and intervention points. For this, we produced a unique model of perimenopausal CAA by inducing chemical AOF in young female mice expressing a human vasculotropic mutant amyloid β precursor protein and conducted anatomical and behavioral measures close to the onset of both ovarian decline and CAA. Using this approach, we found signs of increased reactive astrocytes in select regions of the hippocampus. The heightened activity of astrocytes was accompanied by elevated accumulation of hippocampus Aβ, but not paralleled by increased signs of microglial activation, suggesting that early peri-AOF is associated with select effects on glial cell subtypes. Behaviorally, despite signs of impaired memory in the intact female SWDI mice, peri-AOF did not exacerbate cognitive dysfunction at disease onset in SWDI mice. The latter findings suggest that the increased signs of hippocampal astrocyte reactivity and Aβ deposition associated with peri-AOF have not yet passed a threshold that leads to further deleterious effects on behavior. This points to the possibility that an early stage of ovarian decline may be a stage of increasing hippocampal vulnerability, and thus a window of opportunity to moderate the damaging effects of cerebrovascular dysfunction associated with cerebral amyloid angiopathy and ovarian failure before progressing to a more severe degree of neurovascular compromise and cognitive impairment.

In the present study, we utilized a model of AOF that recapitulates the hormonal changes seen during the progression of human menopause. This approach results in a stage of irregular and extended estrous cycles and erratically fluctuating estrogen levels that correspond to human perimenopause (Van Kempen et al., 2014; Brooks et al., 2016; Marques-Lopes et al., 2017), but with limited effects on peripheral tissues or brain areas inside and outside the blood–brain barrier (Van Kempen et al., 2014). Critically, this method also provides for the induction of ovarian dysfunction in mice at particular developmental time points, including young animals, thus allowing for the isolation of the effects of gonadal hormone signaling from other factors like aging. Thus, the present finding of increased hippocampal GFAP and Aβ in peri-AOF CAA mice indicates that, in the context of cerebral amyloidosis, irregular hormonal cycling alone is capable of inducing detectible increases in astrocyte activation in a brain region critical for cognitive processes that are adversely affected during dementia.

The hippocampus is both structurally and functionally demarcated. For example, infragranular and subgranular zones of the DG contain cells that undergo adult neurogenesis (Araki et al., 2021). In the CA1, the SLM receives entorhinal and thalamic afferents and contains interneurons important for rhythmic synchronization of pyramidal cells involved in mnemonic processes (Chapman and Lacaille, 1999; Vu et al., 2020) and affect neuronal protection and survival (Lana et al., 2021). The CA1 SO contains a variety of inhibitory interneurons (Hardy et al., 2021), in addition to the axons (Arszovszki et al., 2014) and basal dendrites (Bannister and Larkman, 1995; Rigby et al., 2023) of principle cells and receives inputs from the entorhinal cortex (Takács et al., 2012; Bell et al., 2021), local (Takács et al., 2012; Huh et al., 2016; Bell et al., 2021) or commissural (Supèr and Soriano, 1994) principle hippocampal neurons, and from the medial septal nucleus (Yamada and Jinno, 2015), the site of cholinergic neurons (Müller and Remy, 2018). The CA2/CA3a regions are innervated by the hypothalamic supramammillary nucleus believed to participate in theta rhythms influencing encoding of new memories (Jones and McHugh, 2011). However, little is known about the role of early ovarian failure on neuroinflammation across hippocampal subregions in the context of early-stage amyloidosis.

In the present study, densitometric analysis of GFAP was performed across hippocampal subregions. It was found that only at the caudal level of the CA1 within the SO field were GFAP levels increased in peri-AOF SWDI mice. In contrast, GFAP was elevated in SWDI mice in the caudal SUB and in the rostral CA1 SO region of SWDI mice, independent of AOF status. These findings indicate that the SO region of the caudal CA1 exhibits increased astrocytic activity in the context of CAA and early ovarian failure, whereas other regions of the hippocampus (caudal SUB and rostral SO) show signs of astrocyte activity during CAA only. These results suggest that the timing of GFAP expression in SWDI mice varies by hippocampal subregion, which is further influenced by early ovarian failure. It should also be noted that, for the most part, increases in GFAP were paralleled by increases in Aβ. For example, in the case of intact SWDI mice, higher GFAP was accompanied by increased Aβ in the rostral CA1 SO and caudal SUB of intact SWDI mice, although this was not matched by a further increase in GFAP in peri-AOF mice.

Functionally, the CA1 is a critical part of both the monosynaptic and trisynaptic hippocampal circuits that play essential roles in hippocampal signal processing, learning and memory (Basu and Siegelbaum, 2015; Stepan et al., 2015). The CA1 is highly vulnerable to dementia-associated neuropathological processes and is among the earliest to show signs of tissue volume decline/neurodegeneration during disease progression (Su et al., 2018; Dounavi et al., 2020; McKeever et al., 2020). Neuropathological and degenerative processes associated with vascular dementia critically involve reactive astrocytes (Price et al., 2018). Indeed, reactive astrocytes have been well-characterized for their roles in neuroinflammatory processes that contribute to neuropathology during brain ischemia, pathogen infection, as well as trauma (Li and Barres, 2018; Matias et al., 2019) and may promote neural stress and neurodegeneration (Arevalo et al., 2013; Spangenberg and Green, 2017). Astrocytes form a critical component of both the neurovascular unit as well as the tripartite synapse, that are critical for maintaining cerebral function. and which show dysfunction in the hippocampus in models of cerebrovascular disease (Sompol et al., 2023) and dementia (Liu et al., 2018). Beyond neurovascular function, astrocytes are also involved in the clearance of Aβ (Wyss-Coray et al., 2003; Kantarci et al., 2016; Davis et al., 2021), but may also contribute to Aβ production as well (Frost and Li, 2017). Further, astrocytes, along with pericytes and vascular endothelial cells, contribute to BBB formation to maintain brain homeostasis, which is dysregulated by Aβ (Wang et al., 2021). Thus, astrocytes may contribute to CAA-associated hippocampal pathology by distinct mechanisms.

The select increase in reactive astrocytes seen in the SO in peri-AOF CAA mice may be related to the actions of estrogen. Estrogen deficiency has been shown to contribute to the reactive astrocytosis associated with models of dementia (Yun et al., 2018), whereas estrogen may protect against reactive astrogliosis in the hippocampus (Bagheri et al., 2013). Specifically, the SO is a site of estradiol binding, and contains perisynaptic astrocyte profiles that express immunolabeling for estrogen receptor alpha (Towart et al., 2003), which may mediate protective effects against Aβ-mediated pathology (Carbonaro et al., 2009; Hwang et al., 2015). Thus, the increased activity of SO astrocytes at peri-AOF during early-stage CAA may reflect the dysregulation of both estrogen and cerebrovascular function associated with Aβ and may provide the conditions that lead to more severe neuropathology with the progression of ovarian failure and CAA.

The increase in glial reactivity and Aβ in intact SWDI mice was associated with select behavioral outcomes. When tested in the Y-maze or the novel object recognition tasks, SWDI mice showed a deficit in cognitive behavior. However, VCD-treated mice performed at a level comparable to oil-injected animals, indicating that peri-AOF did not result in further impairment in alternation memory. In contrast, neither genotype nor VCD treatment impacted spatial memory as measured in the Barnes maze. The lack of a behavioral effect in the latter paradigm even in CAA mice may be due to the exclusive testing of female mice at a young age and requires further time-course investigation.

## 5 Summary and implications

We performed a granular anatomical analysis of neuroinflammation in the hippocampus in a model of early perimenopausal amyloidosis and revealed that specific hippocampal regions show widely differing susceptibilities to astrocyte activation in response to CAA alone or the combination of CAA and peri-AOF. In particular, peri-AOF coupled with CAA was associated with a select increase in astrocyte activation in the caudal CA1 SO field. Whether changes in estrogen signaling at peri-AOF may specifically intersect with astroglial function or dysfunction to promote early CAA is unknown. Moreover, whether this vulnerability at peri-AOF leads to cascading neuroinflammatory pathology in connected CA1 targets or with progressive cognitive dysfunction at later stages of AOF and amyloidosis awaits future time-course studies.

More generally, temporal factors are emerging as an important consideration in dementia therapeutics. This is highlighted by evidence that Aβ monoclonal antibodies (Sims et al., 2023) given early in Alzheimer’s dementia may be more efficacious compared to use at later stages. Temporal factors have also been noteworthy in the debate about dementia therapeutics in menopausal women. The timing hypothesis posits that hormone replacement started soon after menopause onset reduces the risk of AD dementia (Henderson et al., 2005; Whitmer et al., 2011; Mills et al., 2023). The existence of temporal constraints on the efficacy of hormone replacement is supported by a growing body of literature including findings that perimenopausal hormone therapy protects hippocampal function and structure (Berent-Spillson et al., 2010; Maki et al., 2011), including the volume of the CA1 region (Pintzka and Håberg, 2015). Additionally, other data is consistent with the protective effects of early hormone therapy on dementia risk (Kantarci et al., 2016; Sung et al., 2022; Saleh et al., 2023). In this context, our model of early perimenopausal CAA may have basic and preclinical value as a method to investigate the mechanisms of potential ameliorative effects of hormone replacement, which can be further expanded to encompass other forms of dementia. Ultimately, such findings may provide a better understanding of how hormone dysregulation influences hippocampal health and dementia liability in women across menopause.

## Data availability statement

The raw data supporting the conclusions of this article will be made available by the authors, without undue reservation.

## Ethics statement

The animal study was approved by Weill Cornell Medicine Institutional Institutional Animal Care and Use Committee. The study was conducted in accordance with the local legislation and institutional requirements.

## Author contributions

JP: Conceptualization, Data curation, Formal analysis, Writing – original draft, Writing – review & editing. RM: Conceptualization, Formal analysis, Supervision, Writing – review & editing. LP: Conceptualization, Writing – review & editing. FY: Data curation, Formal analysis, Investigation, Writing – review & editing. GS: Data curation, Formal analysis, Investigation, Writing – review & editing. RW: Investigation, Supervision, Writing – review & editing. WT: Investigation, Visualization, Writing – review & editing. TM: Conceptualization, Formal analysis, Funding acquisition, Supervision, Writing – original draft, Writing – review & editing, Investigation, Project administration. MG: Conceptualization, Formal analysis, Funding acquisition, Supervision, Writing – original draft, Writing – review & editing.
